# Chronic Cadmium Treatment Promotes Oxidative Stress and Endothelial Damage in Isolated Rat Aorta

**DOI:** 10.1371/journal.pone.0068418

**Published:** 2013-07-12

**Authors:** Camila C. P. Almenara, Gilson B. Broseghini-Filho, Marcus V. A. Vescovi, Jhuli K. Angeli, Thaís de O. Faria, Ivanita Stefanon, Dalton V. Vassallo, Alessandra S. Padilha

**Affiliations:** 1 Department of Physiological Sciences, Federal University of Espirito Santo, Vitoria, Espírito Santo, Brazil; 2 Health Science Center of Vitoria - EMESCAM, Vitoria, Espírito Santo, Brazil; 3 Departament of Chemistry, Federal University of Espirito Santo, Vitoria, Espírito Santo, Brazil; Case Western Reserve University, United States of America

## Abstract

Cadmium is a highly toxic metal that is present in phosphate fertilizers, and the incidence of cadmium poisoning in the general population has increased, mainly due to cigarette smoking. Once absorbed, cadmium accumulates in the tissues, causing harmful effects including high blood pressure, endothelial damage and oxidative stress. Oxidative stress is known to efficiently produce oxidized low-density lipoprotein and consequently atherosclerosis, mainly in the aorta. However, the mechanisms through which endothelial damage is induced by cadmium have not been elucidated. Thus, the aim of this study was to investigate the effects of this metal in the isolated aorta and the possible role of oxidative stress. Rats received 100 mg.L^−1^ cadmium chloride (CdCl_2_) in the drinking water or distilled water alone for four weeks. The pressor effect of cadmium was followed throughout the exposure period by tail plethysmography. At the end of the fourth week, the blood cadmium content was established, and the vascular reactivity of the isolated aorta to phenylephrine, acetylcholine and sodium nitroprusside was analyzed in the context of endothelium denudation and incubation with L-NAME, apocynin, losartan, enalapril, superoxide dismutase (SOD) or catalase. We observed an increased response to phenylephrine in cadmium-treated rats. This increase was abolished by catalase and SOD incubation. Apocynin treatment reduced the phenylephrine response in both treatment groups, but its effect was greater in cadmium-treated rats, and NOX2 expression was greater in the cadmium group. These results suggested that cadmium in blood concentrations similar to those found in occupationally exposed populations is able to stimulate NOX2 expression, contributing to oxidative stress and reducing NO bioavailability, despite enhanced eNOS expression. These findings suggest that cadmium exposure promotes endothelial damage that might contribute to inflammation, vascular injury and the development of atherosclerosis.

## Introduction

Cadmium (Cd) is a toxic metal that is commonly used in the production of polyvinyl chloride (PVC), pigments and batteries. Furthermore, its presence in phosphate fertilizers contributes for the increased incidence of cadmium poisoning in the general population, since leaf vegetables, oilseeds and tobacco accumulate high levels of cadmium from soil. Thus, food and smoking are the main sources of non-occupational exposure to cadmium [1]. Tabacco smokers have approximately three times the cadmium blood content than nonsmokers (1.58 µg.L^−1^ for smokers vs 0.47 µg.L^−1^ for nonsmokers) [2]. This content of cadmium has been associated with an increased risk of hypertension and related diseases in smokers [3].

Once absorbed, cadmium is reported to accumulate in several tissues, mainly in the kidneys and liver [4], which might cause harmful effects including renal dysfunction [5], pulmonary edema [6], cancer [7]–[9] and cardiovascular disease [3]. Furthermore, several experimental and clinical studies have associated cadmium and high blood pressure [5], [10]. Reports have highlighted that endothelial damage might be an important process for the development of high blood pressure induced by cadmium [11], [12]. Yoopan et al (2009) showed that chronic exposure to cadmium (10 e 50 ppm) decreased the relaxation response to acetylcholine in aortic ring but did not alter the response to sodium nitroprusside, suggesting impaired endothelial function after cadmium exposure [12]. Other study suggested that cadmium *in vitro* exposure increase both relaxation and contratile responses [13]. Some authors demonstrated that the endothelial damage induced by cadmium (1 mg/kg/day for 14 or 15 days) was associated with increased oxidative stress [14], [15].

Oxidative stress is known to efficiently produce oxidized low-density lipoprotein and consequently cause atherosclerosis [16]. Advanced glycation end products are generated, and the subsequent recruitment of inflammatory cells maintains vascular injury [17]. One of the major sites that develop atherosclerosis due to oxidative stress is the aorta. The fact that the endothelium is affected by low concentrations of heavy metals such as mercury, lead and cadmium, even at levels below the reference values, highlights the importance and the need to better understand the mechanisms by which these metals promote the development of cardiovascular disease [18]–[20].

However, the mechanisms by which endothelial damage is induced by cadmium have not been clearly elucidated. Moreover, there are no reports showing that vascular effects are induced by low concentration of cadmium, similar to those found in the blood after occupational exposure (5–50 µg.L^−1^) [1]. In the present study, we exposed rats to a low concentration of cadmium chloride (100 mg.L^−1^) in the drinking water for 4 weeks to investigate whether the effects of this metal on the isolated aorta could explain some of the mechanisms involved in the cadmium pressor effect.

## Materials and Methods

### Animals

Male Wistar rats (N = 90) weighing 190–210 g were used for this study. All experiments were conducted in accordance with the Guide for the Care and Use of Laboratory Animals and were approved by the Ethics Committee of the Federal University of Espirito Santo (027/2011 CEUA-UFES). During treatment, rats were housed at a constant room temperature, humidity and light cycle (12∶12 h light-dark), had free access to water and were fed standard chow ad libitum. Rats were divided into two groups. The control rats (N = 45) received distilled water as drinking water, whereas the cadmium-exposed rats (N = 45) received distilled water containing CdCl_2_ (100 mg.L^−1^) continuously for four weeks. The animals were weighed prior to and at the last day of the cadmium exposure.

### Blood Pressure Measurements

Systolic blood pressure was measured weekly in conscious rats using noninvasive tail-cuff plethysmography (IITC Life Science, Inc.). Conscious rats were restrained for 5–10 min in a warm and quiet room and conditioned to cuff inflation-deflation cycles prior to the recordings. Systolic blood pressure was measured, and the mean of three measurements was recorded as previously described [21].

### Blood Cadmium Level Measurements

At the end of the four weeks of cadmium exposure, the rats were anesthetized with sodium thiopental (50 mg.kg^−1^, i.p.), and whole blood was collected. The cadmium concentrations in the samples of whole blood were measured in duplicate by atomic fluorescence spectrometry (model: AAS5 EA with graphite furnace, Carl Zeiss, Germany) at the Chemistry Department of the Federal University of Espirito Santo.

### Isolated Rat Aorta Preparation

Thoracic aortas were carefully dissected out and cleaned of fat and connective tissue. For vascular reactivity experiments, the aortas were divided into cylindrical segments of 4 mm in length (5 to 6 aortic rings each). For the analysis of gp91phox (NOX2) and SOD Cu/Zn protein expression, arteries were rapidly frozen in liquid nitrogen and kept at −70°C until the day of analysis.

#### Vascular reactivity measurements

Segments of thoracic aorta (4 mm in length) were mounted in an isolated tissue chamber containing Krebs-Henseleit solution (NaCl 118 mM; KCl 4.7 mM; NaHCO_3_ 23 mM; CaCl_2_ 2.5 mM; KH_2_PO_4_ 1.2 mM; MgSO_4_ 1.2 mM; glucose 11 mM and EDTA 0.01 mM ), gassed with 95% O_2_ and 5% CO_2_ (pH 7.4) and maintained at a resting tension of 1 g at 37°C. Isometric tension was recorded using an isometric force transducer (TSD125C, CA, U.S.A) connected to an acquisition system (MP100A, BIOPAC System, Inc., Santa Barbara, CA, U.S.A).

After a 45-min equilibration period, all aortic rings were exposed twice to 75 mM KCl (30 min), first to check their functional integrity and again to assess the maximal developed tension. Then, the endothelial integrity was tested with acetylcholine (10 µM) in segments that were previously contracted with phenylephrine (1 µM). Relaxation equal to or greater than 90% was considered to demonstrate the functional integrity of the endothelium. After a washout period (30 min), increasing concentrations of phenylephrine (0.1 nM to 0.3 mM) were applied. A concentration–response curve to this agonist was obtained, and tension was measured once a plateau was attained.

The influence of the endothelium on the response to phenylephrine was investigated by mechanically removing the tissue (performed by rubbing the lumen with a needle). The absence of endothelium was confirmed by the inability of 10 µM acetylcholine to produce relaxation. Then, the role of endothelium-derived vasoactive factors in the phenylephrine-elicited contractile response was investigated. The effects of the following drugs were evaluated: a nonspecific NOS inhibitor (N-nitro-L-arginine methyl ester, or L-NAME, 100 µM); an enzyme scavenger superoxide anion, (superoxide dismutase, or SOD, 150 U.mL^−1^); an NADPH oxidase inhibitor (apocynin, 0.3 mM); a scavenger of hydrogen peroxide (catalase, 1000 U.mL^−1^); an angiotensin converting enzyme inhibitor (enalapril, 10 µM); and an angiotensin II type 1 receptor antagonist (losartan, 10 µM). These drugs were added 30 min before the generation of the phenylephrine concentration–response curves.

In another set of experiments, after the 45-min equilibration period, aortic rings were contracted with phenylephrine (1 µM) until a plateau was reached (approximately 15 min), and concentration-response curves to acetylcholine (0.01 nM to 30 µM) or sodium nitroprusside (0.01 nM to 30 µM) were obtained for both groups.

### Nitric Oxide Release

Nitric oxide release was measured as previously described [22]. The segments of thoracic aorta artery was dissected and equilibrated for 60 min in HEPES buffer (in mmol·L-1: 119 NaCl; 20 HEPES; 1.2 CaCl2; 4.6 KCl; 1 MgSO4; 0.4 KH2PO4;5 NaHCO3; 5.5 glucose; 0.15 NaH2PO4; pH 7.4) at 37°C; arteries were then incubated with the fluorescent probe 4,5-diaminofluorescein (2 mmol·L-1) for 1 h and the medium was collected to measure basal NO release. The fluorescence of the medium was measured at room temperature using a spectrofluorometer (LS50 Perkin Elmer instruments, FL WINLAB Software) with excitation wavelength set at 492 nm and emission wavelength at 515 nm. Blank measurement samples were similarly collected but without arteries to subtract background emission. The amount of NO released was expressed as arbitrary units·mg-1 tissue.

### Western Blot Analysis

Proteins from homogenized arteries were separated by 10% SDS-PAGE. Proteins were transferred to nitrocellulose membranes that were then incubated overnight with mouse monoclonal antibodies to either anti-superoxide dismutase Cu/Zn (Cu/ZnSOD, 1∶1000, Sigma, Aldrich, Germany), endothelial nitric oxide synthase (eNOS, 1∶250; Transduction Laboratories, Lexington, UK) or anti-NADPH subunit (gp^91phox^, 1∶1000; Transduction Laboratories, Lexington, UK). After washing, the membranes were incubated with anti-mouse immunoglobulin antibody conjugated to horseradish peroxidase (1∶5000, StressGen, Victoria, Canada). After thorough washing, immunocomplexes were detected using an enhanced horseradish peroxidase/luminal chemiluminescence system (ECL Prime, Amersham International, Little Chalfont, UK) and film (Hyperfilm ECL International). The signals on the immunoblot were quantified with the National Institutes of Health Image V1.56 computer program. The α-actin expression on the same membrane was detected using a mouse monoclonal antibody (1∶5000, Sigma, USA) as a loading control.

### Statistical Analyses

The vasoconstrictor responses induced by phenylephrine were normalized to the contraction induced by 75 mM KCl and expressed as a percentage of this contraction. The vasodilator responses are expressed as the percentage of the previous contraction. For each concentration-response curve, the maximum effect (Rmax) and the concentration of agonist that produced one-half of Rmax (EC50) were calculated using nonlinear regression analysis (GraphPad Prism Software, San Diego, CA). The sensitivity of the agonists was expressed as pD2 (-log EC50). To compare the effects of L-NAME and endothelium denudation on the contractile response to phenylephrine, the differences in the area under the concentration-response curves (dAUC) for phenylephrine in control and experimental situations were calculated. AUCs were calculated from the individual curve plots (GraphPad Prism Software), and differences are expressed as the percentage of the AUC of the corresponding control. These values indicate whether the magnitude of the effect of each treatment is different in the control and cadmium-exposed rats. Protein expression data are expressed as the ratio between signals corresponding to the studied protein and α-actin on the immunoblot.

The results are expressed as the means ± SEM of the number of rats studied; differences were analyzed using Student’s t-test or two-way ANOVA followed by a Bonferroni test. P<0.05 was considered significant.

### Drugs and Reagents

Cadmium chloride (CdCl_2_), l-phenylephrine hydrochloride, L-NAME, enalapril maleate, losartan, acetylcholine chloride, sodium pentobarbital, sodium nitroprusside, superoxide dismutase, catalase, and apocynin were purchased from Sigma-Aldrich (St. Louis, USA). All salts and reagents used were of analytical grade and were obtained from Sigma-Aldrich or Merck (Darmstadt, Germany).

## Results

### The Effects of Cadmium Treatment on Body Weight, Blood Cadmium Content and Arterial Systolic Pressure

There was no between-group difference in body weight at the beginning of the treatment period (Control: 196±5 g, n = 19; Cadmium: 197±5 g, n = 19; P>0.05). However, after exposure, the cadmium-treated group had a lower body weight (Control: 334±9 g, n = 19; Cadmium: 294±7 g, n = 19; P<0.05).

**Figure 1:**
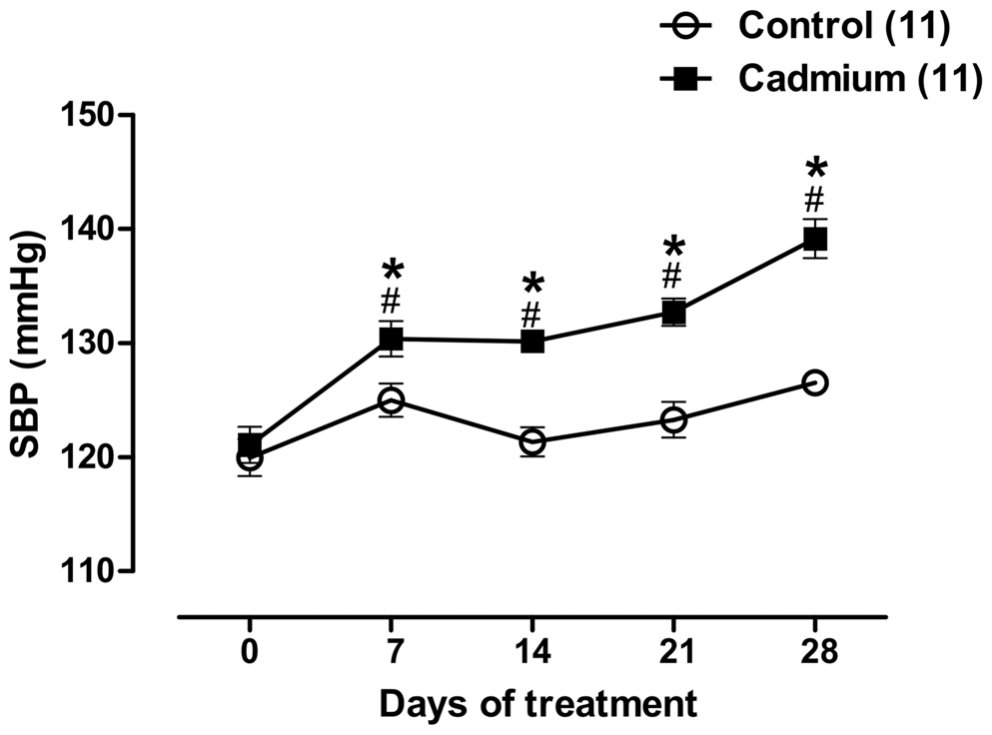
The effects of cadmium exposure on systolic blood pressure in rats. *P<0.05 vs matched exposure day of control group.^ #^P<0.05 vs first day of exposure of Cd-exposure group, determined using two-way ANOVA with Bonferroni’s post-test. The number of animals used is indicated in parentheses.

In rats exposed to four weeks of cadmium treatment, the blood cadmium concentration attained was 40.3±2.0 µg.L^−1^ (n = 11), while in the control group, this level was below 0.9 µg.L^−1^. A significant increase in systolic arterial blood pressure was observed from the first week to the fourth week of cadmium exposure (Figure 1).

### The Effects of Cadmium Treatment on Vascular Reactivity

**Figure 2:**
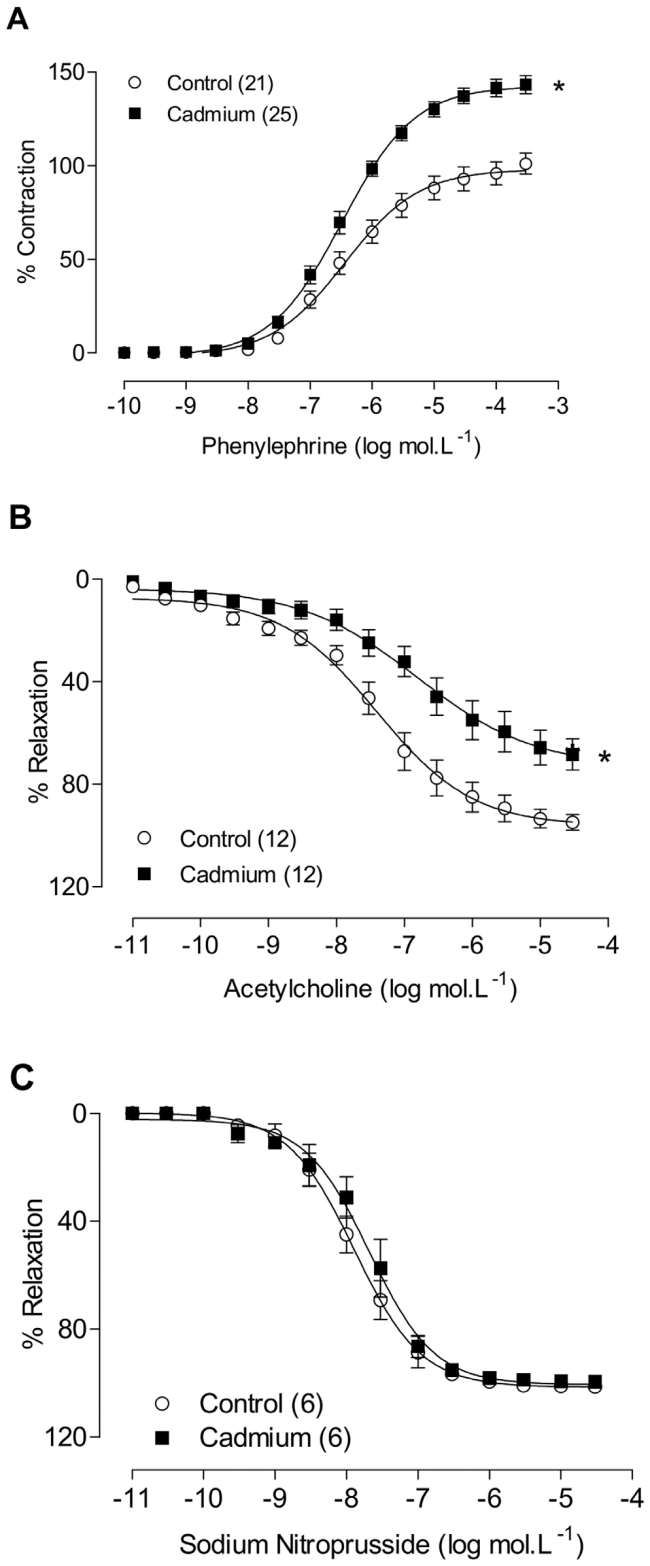
The effects of cadmium exposure on concentration-response curves to phenylephrine (A), acetylcholine (B) and sodium nitroprusside (C). *P<0.05, Student’s t-test. The number of animals used is indicated in parentheses.

Cadmium treatment did not affect the response to KCl (Control: 2.44±0.14 g, n = 29; Cadmium: 2.26±0.12 g, n = 29; P>0.05), but it increased the contractile responses induced by phenylephrine in the rat aortas (Figure 2 A) without changing the sensitivity to this α-1 agonist (Table 1). Cadmium exposure also reduced the concentration-dependent relaxation induced by acetylcholine (Figure 2 B and Table 1). However, the response induced by sodium nitroprusside did not differ between groups (Table 1 and Figure 2 C).

**Table 1:** Sensitivity (pD2) and maximal responses (Emax) to phenylephrine, acetylcholine and sodium nitroprusside in aortic rings from cadmium-treated and control rats.

	Control		Cadmium-treated	
	Emax (%)	pD_2_	Emax (%)	pD_2_
**Phenylephrine**	102±6	6.30±0.16	142±5*	6.52±0.80
**Acetylcholine**	93±4	7.00±0.29	75±6*	6.87±0.19
**Sodium nitroprusside**	103±1	8.06±0.21	105±2	7.63±0.14

The results are expressed as the mean ± SEM; Emax, maximal effect expressed as a percentage of the response induced by 75 mM KCl; pD2, -log one-half Emax. *P<0.05, Student’s t-test.

**Figure 3:**
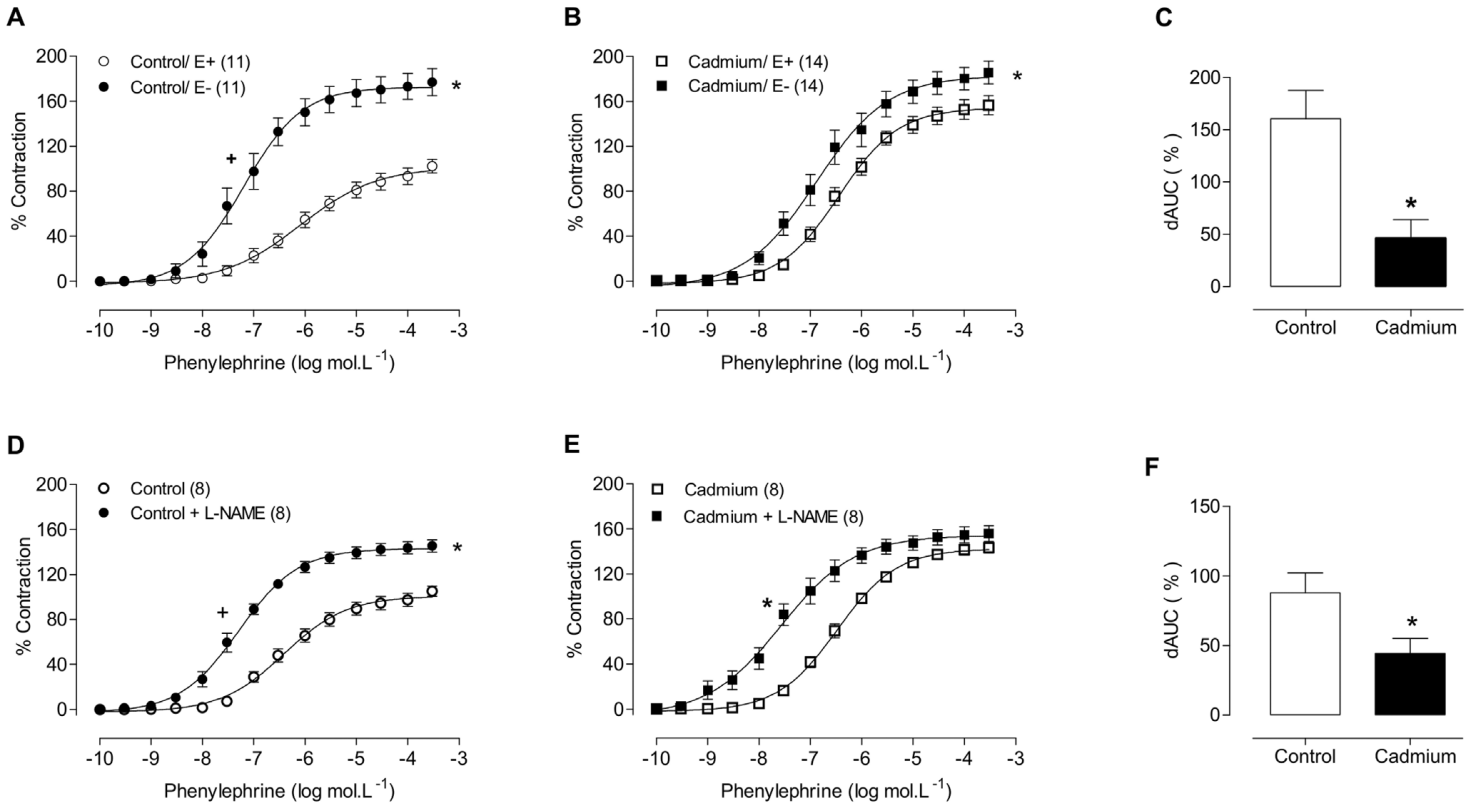
Phenylephrine concentration-response curves after endothelium removal (A, B) or N-nitro-L-arginine methyl ester incubation (L-NAME, 100 µM) (D, E) in the aortic rings of control and cadmium-exposed rats. E-: endothelium-denuded, E+: intact segments. Differences in the area under the concentration-response curves (dAUC) in endothelium–denuded and intact segments (C) and in the presence and absence of L-NAME (F). * and+P<0.05, Student’s t-test. The number of animals used is indicated in parentheses.

**Figure 4:**
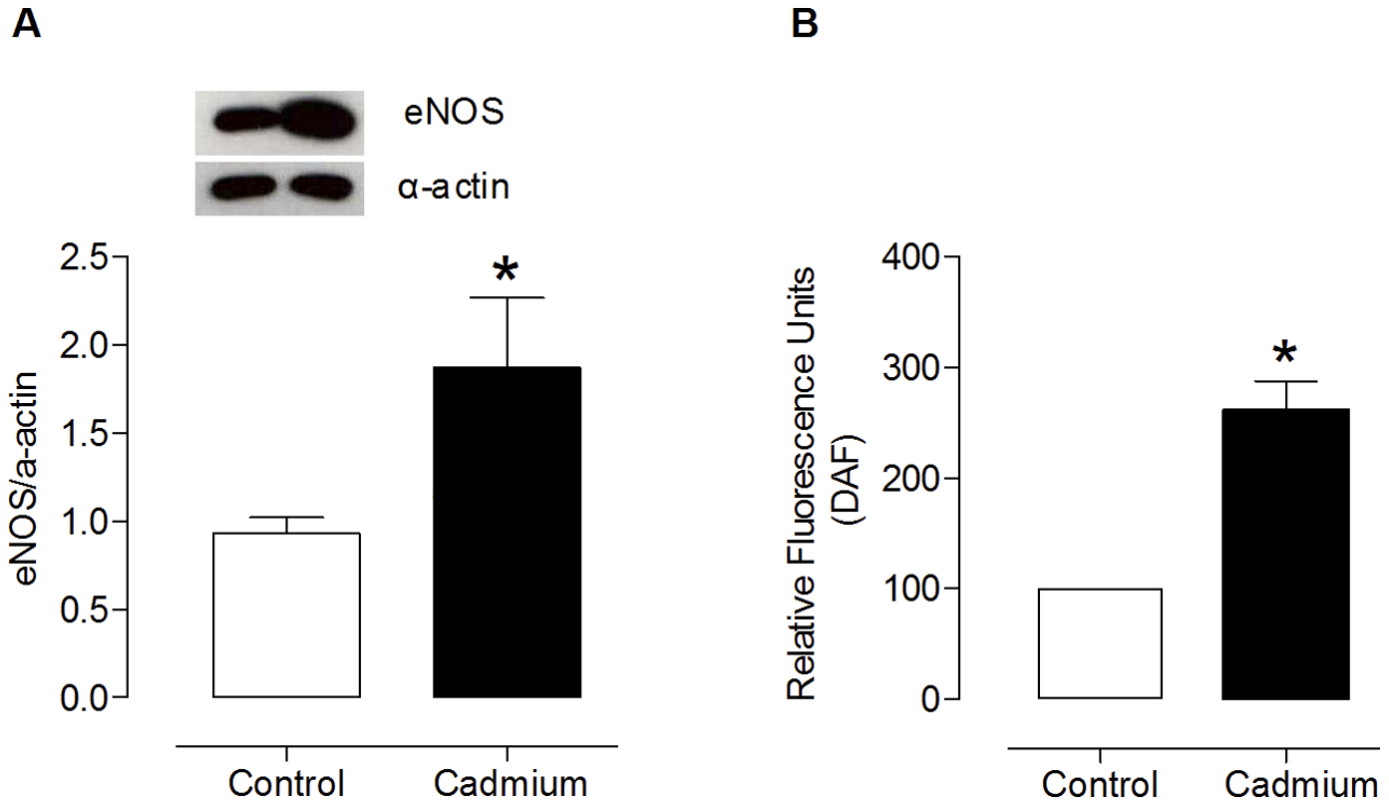
Densitometric analysis of western blot for eNOS protein expression (A) and NO production (B) in aortic rings incubated with DAF (2 mmol·L ^−**1**^ **).** Representative blots are also shown. *P<0.05, by Student’s t-test.

In aortic segments from either group, both endothelium removal and incubation with the NOS inhibitor L-NAME (100 µM) shifted the phenylephrine concentration-response curves to the left. However, this shift was smaller in preparations from cadmium-exposed rats than in control rats, as shown by the dAUC values (Figure 3; Table 2). Furthermore, cadmium exposure increased eNOS protein expression and NO production (measured by DAF) in isolated aortic rings (Figure 4).

**Table 2:** The effects of endothelium removal (E^−^), L-NAME, losartan, enalapril, SOD, catalase and apocynin on the sensitivity (pD2) and maximal response (Emax) to phenylephrine in aortic rings from cadmium-treated and control rats.

	Control		Cadmium-treated	
	Emax%	pD_2_	Emax%	pD_2_
**E+**	102±6	6.30±0.16	142±5	6.52±0.80
**E−**	177±12^#^	7.20±0.13^#^	186±10*	6.84±0.19
**E+/L-NAME**	145±6^#^	7.31±0.13^#^	156±7	7.65±0.26*
**E+/Losartan**	106±11	6.31±0.13	135±9	6.52±0.20
**E+/Enalapril**	104±8	6.41±0.15	140±11	6.36±0.23
**E+/SOD**	104±5	5.95±0.18	115±8*	6.18±0.28
**E+/Catalase**	105±7	6.34±0.13	104±9*	6.51±0.15
**E+/Apocynin**	79±9^#^	6.44±0.10	90±6*	6.64±0.08

The results are expressed as the mean ± SEM; Emax, maximal effect expressed as a percentage of the response induced by 75 mM KCl; pD2, -log one-half Emax. E- endothelium removal, E+ intact endothelium, SOD, superoxide dismutase. P<0.05 *vs* control rats (^#^) and cadmium-treated rats (*), determined using Student’s t-test.

**Figure 5:**
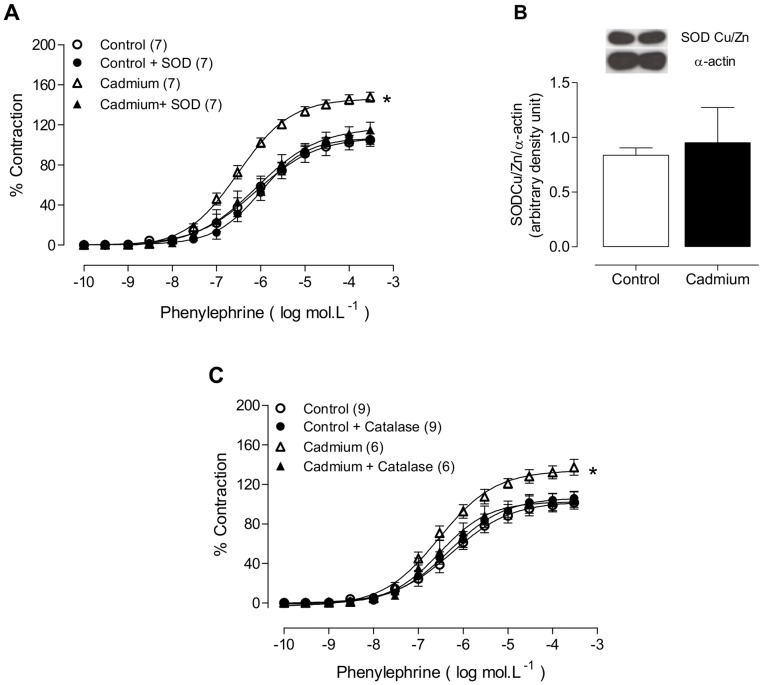
Phenylephrine concentration-response curves in the aortic rings of control and cadmium-exposed rats after SOD (A) or catalase (B) incubation. Densitometric analysis of the western blot to measure the superoxide dismutase Cu/Zn (Cu/ZnSOD) protein expression in the aortic rings of control and cadmium-treated rats (C). Representative blots are also shown. *P<0.05, Student’s t-test. The number of animals used is indicated in parentheses.

To investigate whether the local renin-angiotensin system was involved in the cadmium-induced alterations of vascular reactivity after phenylephrine treatment, angiotensin converting enzyme and AT_1_ receptors were blocked with enalapril (10 µM) and losartan (10 µM), respectively. However, as shown in Table 2, neither drug altered the vasoconstrictor response induced by phenylephrine in aortas from cadmium-treated rats.To determine whether cadmium exposure increased the oxidative stress in the arteries of cadmium-treated rats, the effects on the vasoactive responses of the superoxide anion scavenger SOD and the hydrogen peroxide scavenger catalase were evaluated. As shown in Figure 5 and Table 2, these drugs reduced the vasoconstrictor response induced by phenylephrine in aortas from cadmium-treated rats but not in aortas from untreated rats. However, cadmium exposure did not affect the Cu/Zn SOD protein expression in aortic rings from cadmium-treated rats (Figure 5 and Table 2).

**Figure 6:**
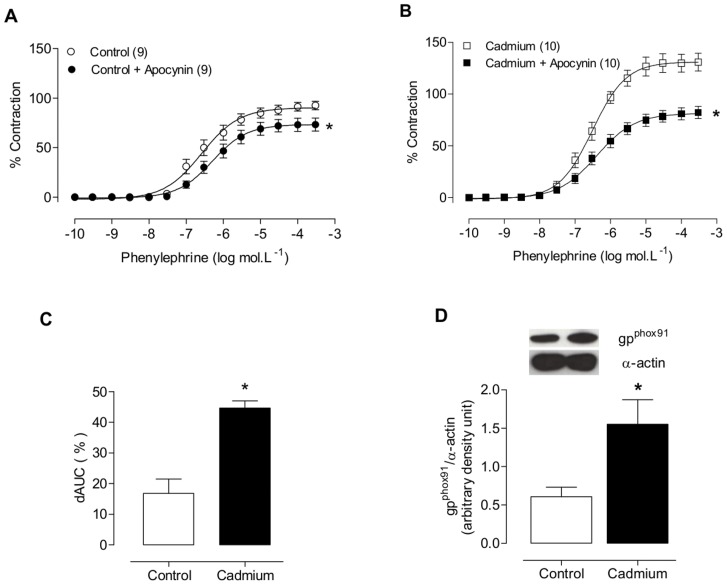
Phenylephrine concentration-response curves in the aortic rings of control (A) and cadmium-exposed (B) rats after apocynin incubation. Differences in the area under the concentration-response curve (dAUC) in the aortic rings of control and cadmium-treated rats after apocynin incubation (C). Densitometric analysis of the western blot to measure the gp^91phox^ protein expression in aortic rings (D). Representative blots are also shown. *P<0.05, Student’s t-test. The number of animals used is indicated in parentheses.

To investigate whether NADPH oxidase was a source of increased superoxide anion production in the aortic rings of cadmium-treated rats, we used apocynin, a NADPH oxidase inhibitor. Apocynin reduced the phenylephrine responses in aortic segments from both groups, but its effect was greater in the cadmium-treated rats (Figure 6A and B). Moreover, cadmium treatment increased the protein expression of gp91^phox^, a NOX2 subunit (Figure 6C).

## Discussion

The major findings in the present study demonstrated that chronic treatment with low concentrations of cadmium, similar to those found in occupationally exposed populations [1], induces endothelial dysfunction in the aorta by decreasing NO bioavailability due to the increased superoxide production caused by an increase in NOX2 activity. These findings suggest that cadmium might contribute to increased blood pressure, as evidenced by a number of treatment effects; for example, vasoconstrictor responses to phenylephrine were increased, NO endothelial modulation was reduced and the endothelium-dependent vasodilator response induced by acetylcholine was also decreased. Meanwhile, the O_2_^−^ scavenger SOD and the NADPH oxidase inhibitor apocynin reduced phenylephrine responses in the aortic rings of cadmium-treated rats. We also observed an increase in the expression of gp91^phox^, a NOX2 subunit, in the vessels of treated rats.

Exposure to cadmium is already recognized to produce toxicological consequences [1]. In the general population, the main sources of intoxication are cigarette smoke and contaminated food [2]. Occupationally exposed workers in smelting, welding, electroplating, refining, pigment production, and battery manufacturing can present cadmium blood concentrations as high as 5 to 50 µg. L^−1^ [1].

In the present study, we observed that chronic low concentration cadmium administration increased the blood cadmium concentration to values similar to those observed in occupationally exposed populations (40.3±2.0 µg.L^−1^), and this concentration was able to increase systolic blood pressure. The cardiovascular toxicity of mercury, cadmium and other divalent metal ions has long been known [19], [23], [24]. We observed that after one week of treatment with cadmium, the systolic blood pressure increased, and this increase was maintained until the end of the experimental period. These results are consistent with previous reports [5], [25], in which high blood and urine cadmium levels were associated with hypertension. However, the underlying mechanisms involved in the development of cadmium-induced high blood pressure at the vascular level are still unclear.

We observed that chronic exposure to cadmium promoted an increased reactivity to phenylephrine in the aortic rings. Moreover, when comparing the dAUC% of aortic rings with and without endothelium, we observed that the magnitude of the response to phenylephrine was lower in the aortic rings of cadmium-treated rats. These results suggest that the ability of the endothelium to negatively modulate the contractile response induced by phenylephrine may be impaired in cadmium-treated rats. Reinforcing this finding, acetylcholine reactivity was reduced in vessels from cadmium-treated rats. Therefore, the increased vascular reactivity to phenylephrine and the concomitant reduction of endothelial modulation associated with reduced acetylcholine reactivity suggest that the treatment with cadmium reduces NO bioavailability [26]. Previous reports have also demonstrated that cadmium exposure reduces the relaxation response to acetylcholine [10], [12]. However, Takahashi et al. [13] showed that the treatment with 10 µM cadmium chloride for 24 hours enhanced contractile responses to phenylephrine and relaxation to acetylcholine in isolated aortic rings of rats. These contradictory results are probably due to differences in the timing and form of cadmium treatment.

To investigate the hypothesis that cadmium might reduce NO bioavailability, we used a non-selective NOS inhibitor, L-NAME. Incubation with L-NAME (100 µM) shifted the phenylephrine concentration-response curves to the left in aortic segments from both groups. However, this shift was smaller in preparations from cadmium-exposed rats than in control rats, suggesting that chronic cadmium exposure can reduce the NO bioavailability. Similar results were reported by Gokalp et al (2009), who reported a significantly greater inhibition of the relaxation response to acetylcholine after L-NAME incubation in the aortic rings of cadmium-hypertensive rats than in control rats, indicating that the cadmium exposure reduces nitric oxide bioavailability [15]. Together, these results demonstrate that cadmium exposure induces endothelial dysfunction in the aorta, thereby reducing the endothelium-induced NO modulation of vasoconstrictor responses.

On the other hand, we observed an overexpression of eNOS in aortic rings from cadmium treated rats. As showed in Figure 4, our results indirectly suggested an increase in H_2_O_2_ (Figure 5) that might increase eNOS protein expression, as reported by Drummond et al [27]. Thereby, the increased eNOS expression could enhance NO production, which was confirmed in treated-aortic rings incubated with fluorescent probe 4,5diaminofluorescein (DAF). However, we did not observe an increase in NO release on phenylephrine contractions in aortic rings of treated-rats. Therefore, we speculate that in presence of phenylephrine, it occurs an increase of ROS release that, in turn, reduces NO bioavailability for smooth muscle relaxation [28]. Therefore, in this condition, NO might act as antioxidant agent.

Given that the superoxide anion interacts with NO-generating peroxynitrite, thereby decreasing NO bioavailability for smooth muscle relaxation [28], we investigated whether oxidative species contribute to the enhanced response to phenylephrine in cadmium-treated rats. To this end, we used a superoxide scavenger enzyme, SOD, and a hydrogen peroxide scavenger enzyme, catalase. Both enzymes reduced the maximal response to phenylephrine to control levels, suggesting that cadmium exposure causes oxidative stress. Cadmium could increase oxidative stress by inducing the generation of ROS, damaging the antioxidant defense systems of cells by depleting GSH and inhibiting SH-dependent enzymes, or interfering with some essential metal(s) needed for antioxidant enzyme activities [19], [29]–[31].

NADPH oxidase is the main source of oxidative stress in the cardiovascular system [32]. The NADPH oxidase (NOX) family includes a number of isoforms, with NOX2 as a prototype. NOX2 is present in endothelial cells and is usually activated by shear stress and angiotensin II [33]. To verify whether NOX has a putative role in mediating the effects of cadmium exposure, we used a non-selective NADPH oxidase inhibitor, apocynin. Apocynin treatment promoted a greater decrease in the vasoconstrictor response to phenylephrine in the aortic rings of cadmium-treated rats than in control rats. This finding suggested that cadmium exposure led to increased NADPH activity, suggesting that the oxidative stress induced by this enzyme could reduce NO bioavailability. In addition, we found that cadmium exposure increased NOX2 expression in the aorta, as previously shown by Ferramola et al in heart tissue [34].

Finally, the oxidative stress induced by cadmium through an increase in lipid peroxidation might also be responsible for the minor weight gain of exposed animals, as previously reported [35]–[37].

The interpretation of these results obtained thus far must consider two limitations. The first limitation pertains to the cadmium concentration we used in this study. Although this is a concentration similar to those found in the blood after occupational exposure (5–50 µg.L^−1^) [1], it does not represent the “safe” value for human beings. We used this cadmium concentration to begin to understand its effects; in the future, lower concentrations should be used. The second limitation is that it is not possible to compare the present results with those in other vascular beds, such as resistance vessels. However, the results of the present study provide guidance for further studies using much lower cadmium concentrations, such as those found in smokers, to better elucidate the cardiovascular effects of this metal.

In summary, our results suggest that cadmium, in concentrations similar to those found in occupationally exposed populations, is able to stimulate NOX2 expression, inducing superoxide anion production by NADPH oxidase and thus reducing NO bioavailability, despite enhence eNOS expression. Taken together, these results suggest that cadmium exposure could promote endothelial damage that might in turn contribute to inflammation, vascular injury and the development of atherosclerosis, rendering the cadmium-induced vascular hyperreactivity to phenylephrine. Thus, our results support the hypothesis that individuals who are occupationally exposed to cadmium might have an increased risk of hypertension and other related disorders.
